# Application and Development of Modern 3D Printing Technology in the Field of Orthopedics

**DOI:** 10.1155/2022/8759060

**Published:** 2022-02-15

**Authors:** Binglong Li, Meng Zhang, Qunshan Lu, Baoqing Zhang, Zhuang Miao, Lei Li, Tong Zheng, Peilai Liu

**Affiliations:** ^1^Department of Orthopedics, Qilu Hospital of Shandong University, Jinan, 250012 Shandong, China; ^2^Shandong University Cheeloo College of Medicine, Jinan, 250100 Shandong, China; ^3^Department of Orthopedics and Trauma, Peking University People's Hospital, Beijing 100044, China

## Abstract

3D printing, also known as additive manufacturing, is a technology that uses a variety of adhesive materials such as powdered metal or plastic to construct objects based on digital models. Recently, 3D printing technology has been combined with digital medicine, materials science, cytology, and other multidisciplinary fields, especially in the field of orthopedic built-in objects. The development of advanced 3D printing materials continues to meet the needs of clinical precision medicine and customize the most suitable prosthesis for everyone to improve service life and satisfaction. This article introduces the development of 3D printing technology and different types of materials. We also discuss the shortcomings of 3D printing technology and the current challenges, including the poor bionics of 3D printing products, lack of ideal bioinks, product safety, and lack of market supervision. We also prospect the future development trends of 3D printing.

## 1. Introduction

In the past few years, the incidence of musculoskeletal disorders caused by trauma and disease has increased year by year, which requires us to adopt more effective strategies to meet this need. 3D printing has developed rapidly as a new industrial technology recently. Unlike traditional subtractive manufacturing, this additive manufacturing method is widely used in aerospace, vehicle manufacturing, energy storage, bioengineering, medical devices, and dentistry [1–3]. 3D printing, also known as additive manufacturing (AM), refers to the use of print heads, nozzles, or other printing technologies to produce powdery metals, plastics, biological materials, and other adhesive materials by layer-by-layer printing based on digital models [4]. At present, technologies such as vat photopolymerization, fused deposition modeling, powder bed fusion, and bioprinting are widely used [5]. Metals, ceramics, polymers, and composites are widely used in 3D printing. This article systematically summarizes the progress in orthopedic applications from the aspects of 3D printing technology and materials and discusses and prospects the future development trends and prospects of the field.

## 2. 3D Printing Techniques

### 2.1. Vat Photopolymerization

**Figure 1:**
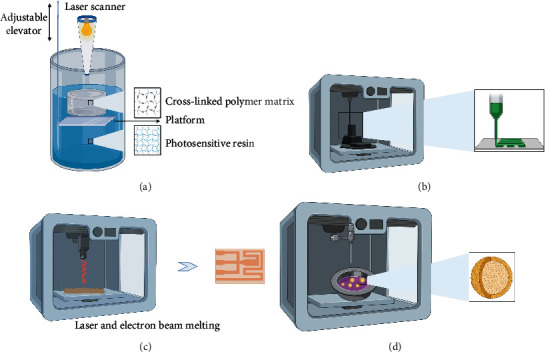
Main 3D printing techniques: (a) vat photopolymerization, (b) fused deposition modeling, (c) selective laser sintering, and (d) bioprinting.

**Figure 2:**
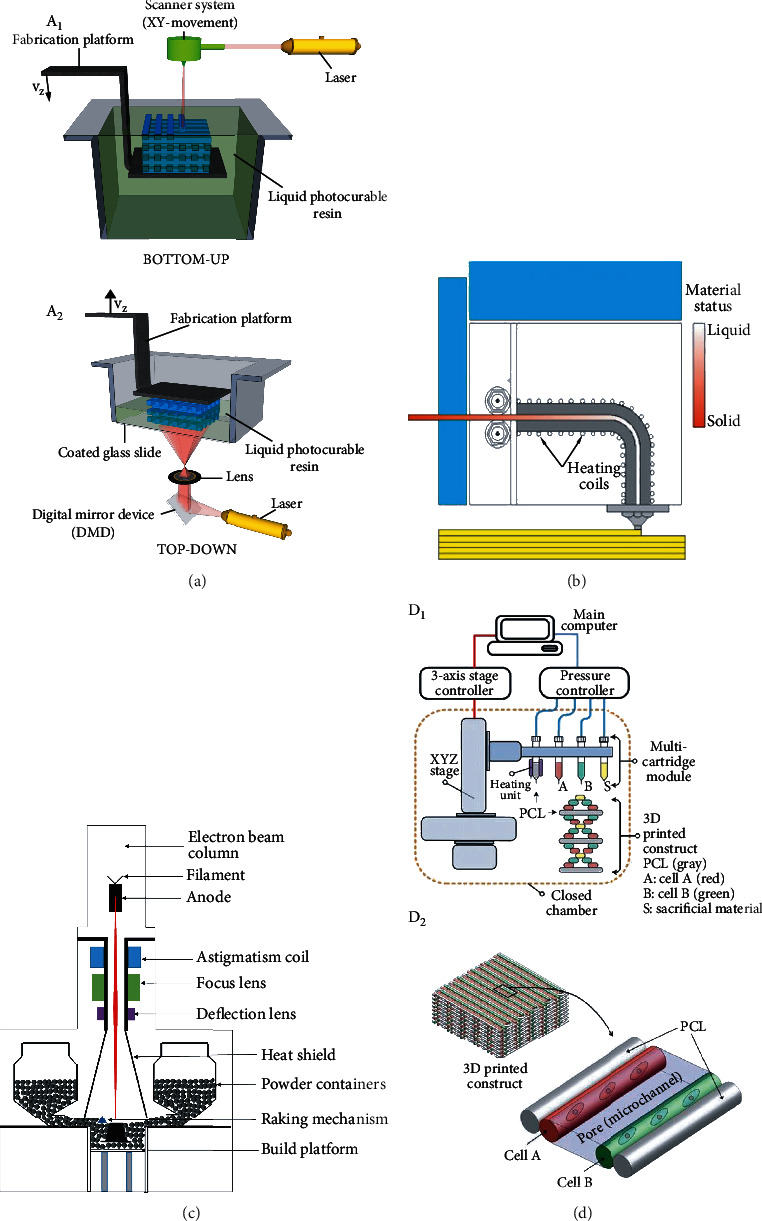
(a) Scheme of bottom-up and top-down stereolithography setups. (a1) The bottom-up setup shown is an example of a system whereby the laser scans the surface for the curing of the photosensitive material. (a2) In the example of the top-down setup, dynamic light projection technology is used to cure a complete 2D layer at once [9]. (b) Fused deposition modeling process [5]. (c) Schematic representation of the EBM process [27]. (d1) Schematic of a bioprinting system and basic patterning of a 3D architecture. (d2) Introduction of basic patterning of 3D architecture including multiple cell-laden hydrogels and supporting PCL polymers [24]. Used with permission from Elsevier and Nature America Publishing.

Vat photopolymerization, also known as stereolithography (SLA), is the first commercial application of 3D printing technology. SLA (Figure 1(a)) starts with the solution in the vat and maps the required pattern on the surface of the solution according to the CAD file. Focusing the ultraviolet rays in a cylinder filled with photosensitive resin, using light propagation chain polymerization, the photosensitive resin is cross-linked on the build plate to form a polymer matrix and then cured layer by layer until the digital 3D object is printed [6–8].  Figure 2(a1) shows the bottom-up scheme, which is the most practical method at present; the top-down scheme shown in Figure 2(a2) has attracted widespread attention. The ultraviolet light from the polymerization of photosensitive materials comes from the bottom of the vat. The direction of platform movement is opposite to the bottom-up approach [9]. According to different photoinitiators, photopolymerization can be divided into two types: free radical and cationic polymerization. The former has a higher reaction speed but faces the challenge of high shrinkage and is prone to curling and warping; the latter rarely results in curling, warping, and shrinking. This is mainly because the former forms a long chain first, and then cross-linking reaction occurs, and the latter starts from a polymer with a cyclic structure, and the newly formed chemical bonds are similar to the original [5].

In SLA, oxygen inhibition of free radical polymerization is a common obstacle to UV polymeric resins. An oxygen-inhibited dead zone (a thin uncured liquid layer) is established between the surface of the cured part and the window so as to realize continuous liquid interface production (CLIP), thereby avoiding the interruption of the processing process [10]. Recently, the dual-curing process has been commercialized in the continuous liquid-phase interface production process to better enhance the surface finish. In addition, due to the improvement of the process, it can also be applied to the manufacture of a wider range of part sizes [11].

### 2.2. Fused Deposition Modeling

Fused deposition modeling is the second commonly used 3D printing technology. The starting material of FDM is a polymer composed of thermoplastic materials. As shown in Figure 1(b), the polymer filaments are fed into the heated print head and nozzles and heated into a molten semisolid form. The molten filaments are rapidly cooled and solidified. The print head squeezes the material on the *x*-*y* axis plane and deposits it layer by layer along the *z* axis on the build plate [12]. A schematic of the FDM process is shown in Figure 2(b) [5]. When facing hollow, suspended, or large inclined structures, additional printing support structures are required [13]. FDM printing requires thermoplastic materials (linear macromolecules), but most pharmaceutical grade materials do not meet the requirements. An improved technology based on FDM, called fused deposition of ceramics (FDC), gets rid of the limitations of materials used [7].

The control of the interlayer adhesion of FDM is complicated, and poor adhesion will affect the mechanical properties of the object, which will lead to delamination. The resolution and layer thickness of the printer are both related to the size of the extruded filaments, increasing the speed while reducing the resolution [14]. Therefore, it is necessary to reasonably weigh the influence of various factors in order to achieve the optimization of printed items. Compared with other 3D printing technologies, FDM has the possibility of multimaterial printing. By using multiple nozzles with different filaments, different materials can be used to print a single object, thereby achieving topological control of different properties of the object [15, 16].

### 2.3. Powder Bed Fusion

Powder bed fusion is a powder-based AM technology that uses high-power laser and electron beam to melt and fix material particles on the build plate to build 3D printed objects. According to different sources of heat energy, powder bed fusion can be divided into laser- and electron beam-based powder bed fusion. The former can be divided into selective laser sintering (SLS) and selective laser melting (SLM), and the latter mainly includes electron beam melting (EBM) [17, 18]. SLS is the most common method. SLS (Figure 1(c)) uses a laser beam to induce the powder to melt.  Figure 2(c) shows the process schematic of EBM technology. The laser does not raise the temperature of the powder to the sintering point but near the sintering point. This sintering step transforms the solid powder into a semiliquid state, and then the platform descends to provide space for the next layer of powder until the top layer of the product is melted. The sintered powder in this process forms the final product, and the unsintered powder serves as a support material, which is then removed by postprocessing. Preheating helps reduce the temperature gradient of the sintered and unsintered parts, thereby eliminating thermal stress [8, 19].

Powder bed fusion has the advantage of being able to manufacture small and complex high-precision parts and a wide range of printing materials. Compared with other printing technologies, an important difference is that the printing environment needs to be oxygen-free to prevent the metal powder from being oxidized at higher temperatures. SLM usually requires nitrogen and argon protective gases, and EBM requires a vacuum environment [20].

### 2.4. Bioprinting

As shown in Figure 1(d), biological 3D printing is a 3D printing technology that uses the living cells, extracellular matrix, biological factors, and biological materials as raw materials and restores its lost functions by constructing highly bionic and biologically active tissue and organ substitutes. Constructing bioscaffolds and finding the optimal bioink are two important issues faced by 3D printing bone tissue. Designing a scaffold requires constructing an ideal bone tissue structure, simulating the microenvironment of bone tissue, and then depositing the bioink loaded with cells and other biocompatible materials into the designed scaffold. When embedding the bioink for bone tissue, the bioink must be embedded on the periphery of the scaffold. This is because when it occurs inside, the concentration of calcium ions will decrease, which is to locate the cancellous bone and the cortical bone. The scaffold must have interconnected pores to reach the center of the scaffold, which is conducive to providing nutrients and growth factors to promote the growth of the bone center [21–23].  Figure 2(d1) shows an integrated tissue-organ printer (ITOP) for printing complex human tissues and organs.  Figure 2(d2) shows the cell-loaded hydrogel and PCL support structure composed of fibrinogen, glycerin, gelatin, and HA [24]. Recently, 3D bioprinting of bone tissue has taken function realization as the printing goal and adopted new printing strategies. “High-precision printing” combines nano/micro-nanotechnology with 3D printing technology to improve printing resolution. “Vascular network printing” develops a bionic bone tissue structure with perfusible endothelialized vascular channels and combines multimaterial printing and vascularization. Some tissues in the body present obvious gradients in the extracellular matrix (ECM) and cell populations at the boundary, such as the osteochondral interface. By adjusting the volume ratio of the two biological inks, “gradient/multifunctional printing” with continuous gradient tissue structure is obtained [25, 26].

## 3. Typical Process of 3D Printing

For individualized customization in orthopedic applications, accurate individual medical image data must be obtained to achieve 3D printing. In the past few decades, with the development of multidetector computed tomography (MDCT) and magnetic resonance imaging (MRI), accurate and high-resolution three-dimensional image data can be quickly obtained. After image postprocessing, multiplane reformatted two-dimensional images and specific three-dimensional images can be generated. Build a computer-aided design (CAD) model and obtain the required products through 3D printing. The conversion of medical image data to 3D printing products is generally divided into 3 steps: image acquisition, image postprocessing, and 3D printing [28, 29].

### 3.1. Image Acquisition

Image acquisition is very important in the 3D printing production process because the quality of printed products depends on the quality of medical images. MDCT and MRI are currently commonly used image acquisition methods. When bones are the region of interest for imaging research, MDCT becomes the preferred imaging method due to its high contrast and simple image postprocessing. When bone tumors invade soft tissues, articular cartilage, and meniscus injuries, MRI is the preferred imaging method. However, artifacts caused by motion during long-time scanning can affect the quality of the image [30]. Other noninvasive imaging techniques can also be used for image acquisition, such as ultrasonic (US), positron emission tomography (PET), and cone beam computed tomography (CBCT). The image data collected by the above methods are usually saved in Digital Imaging and Communications in Medicine (DICOM) format [28].

### 3.2. Image Postprocessing

Image postprocessing is the data reconstruction of the obtained DICOM image, including image segmentation, computer-aided design (CAD), and format conversion [31, 32]. CAD software converts the contour of the region of interest in the image data into a three-dimensional triangular mesh, that is, mesh processing. Irregular curved surfaces will appear in the meshing process, and the tiny triangular plane can produce a smoother surface, which helps to use the triangular surface to approach the shape of the product. CAD information is converted into additive manufacturing file format, Surface Tessellation Language (STL) [33].

### 3.3. 3D Printing

The process of using CAD data generates a three-dimensional object model during 3D printing. The CAD software analyzes the three-dimensional model that the STL file wants to make, and the slicing software slices the model into a series of thin sections. STL files are converted into G-code to control the 3D printer to create 3D products by continuously adding materials to create virtual layers [33]. The technology used for 3D printing has been described in detail in the previous section. The following table (Table 1) summarizes in detail the advantages and disadvantages of different 3D printing technologies and the materials used.

## 4. Materials of 3D Printing

### 4.1. Metals and Alloys

**Figure 3:**
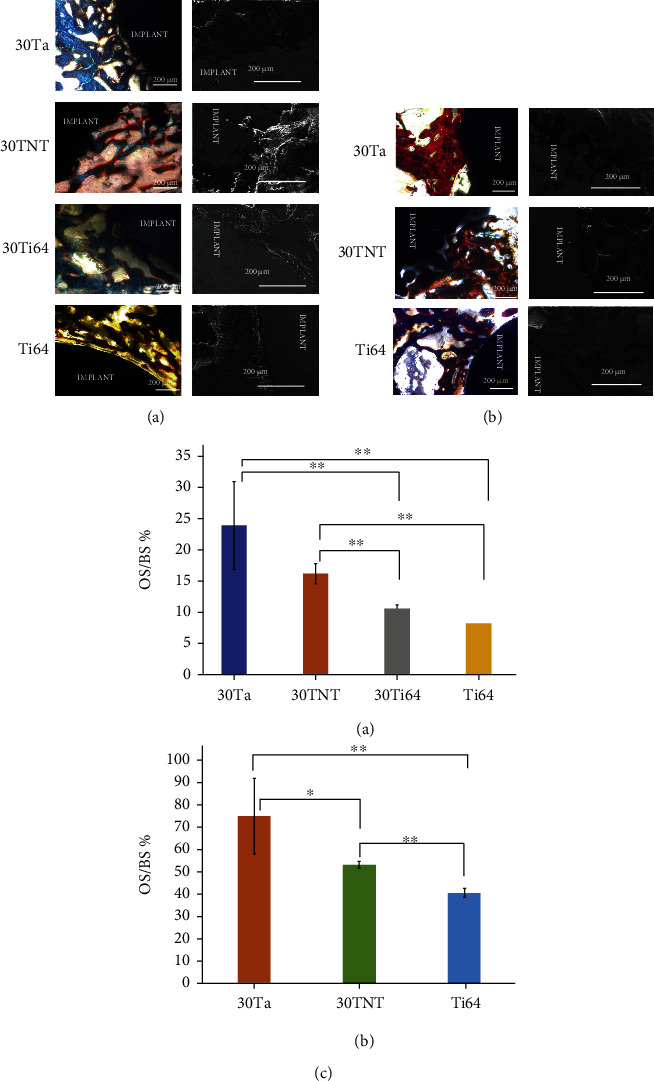
(a) Shows optical and FESEM micrographs of the bone-implant interface 5 weeks postimplantation. (b) Shows optical and FESEM micrographs of the bone-implant interface 12 weeks postimplantation. (c) Shows the histomorphometric plots of OS/BS% for 5 weeks and 12 weeks of histology data, respectively [49]. Used with permission from Elsevier.

Metal has good toughness, bending fatigue resistance, biocompatibility, and excellent processing properties and is currently the most widely used load-bearing implant material. Titanium (Ti), as an important implant material for the weight-bearing parts of orthopedic diseases, usually requires good biocompatibility. Previous studies have shown that Young's modulus of pure titanium is much higher than that of natural bone, which easily leads to stress barriers and prosthesis loosening [2]. To further improve its performance, tantalum (Ta) and niobium (Nb) are usually added as stable materials. Ti is modified by 3D printing technology to create a porous structure to enhance the ability of osteointegration. Studies have shown that the porous titanium alloy can promote collagen production, increase the level of alkaline phosphatase, and promote the proliferation, differentiation, and mineralization of preosteoblasts [45]. The titanium alloy prepared by SLM has a porous lattice structure. After being implanted into the human body, a titanium oxide passivation film is formed, which induces the deposition of calcium and phosphorus ions to form apatite and at the same time adsorbs fibronectin and combines with integrin to produce the BMP2, BMP4, and BMP7 osteogenesis environment, showing a certain degree of osteogenesis [46–48]. Bandyopadhyay et al. [49] used laser engineered net shaping to manufacture porous Ta and Ti6Al4V and compared the effects of surface characteristics and material chemistry on early osseointegration in vivo through a rat distal femur model. The 30% porous Ta and 30% porous Ti6Al4V implants have the same effect in achieving early osseointegration. In vivo studies conducted in a rat distal femur model show that volume fraction porosity plays a crucial role in the early osseointegration of porous metal implants. In addition, under the effect of surface modification (titania nanotubes), the host bone interface and the implant are linked well, and new bone or bone-like formation can be seen (as shown in Figure 3).

**Figure 4:**
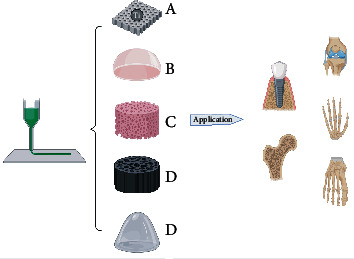
The application of 3D printing in orthopedics.

Ti6Al4V is widely used in orthopedic implants, such as intramedullary nails, due to its good corrosion resistance and high tensile strength. As suggested in Figure 4, porous titanium alloys are widely used in orthopedics. Using a heat treatment process, the surface of Ti6Al4V prepared by SLM is rougher and more hydrophilic, and the texture is more uniform, which enhances the bone bonding ability [50]. This shows that the combination of heat treatment and SLM technology is conducive to customizing customized implants with better biocompatibility and accelerating their application in the field of orthopedics.

CoCrMo is widely used in the implantation of orthopedic prostheses due to its good corrosion resistance and high tensile and yield strength, such as the femoral condyle prosthesis in TKA and femoral stem and acetabular cup in THA [51]. Fernandes et al. studied the metabolism of osteoblasts and endothelial cells in CoCr-rich media and found that the expression of VEGF, alkaline phosphatase, and osteocalcin genes increased significantly, and the shear stress metabolites that respond to intervertebral discs are beneficial to osteogenesis [52]. This provides a new direction for clinically looking for ways to enhance bone healing in the future.

Copper (Cu) can promote metabolism and has antibacterial activity. Duan et al. prepared the Ti-Si-Cu coating in situ by laser, adding different contents of Cu to the coating, and by counting the number of colonies, they found that TS-xC has a good bactericidal ability against Staphylococcus aureus, and the more the Cu content of the coating, the better the antibacterial effect on Staphylococcus aureus [53]. Further research on the antibacterial properties of Cu is of great significance for preventing inflammation caused by prosthesis infection.

Different from the nondegradability of titanium and nickel, magnesium has attracted people's attention as a new type of degradable metal due to its faster corrosion rate than inert metals. Studies [54] have shown that the Mg scaffold prepared by SLM technology can still maintain the mechanical stability of the trabecular bone after four weeks of biodegradation, which indicates that the magnesium scaffold made by additives can help optimize its mechanical properties. With the emergence of new methods and technologies, metal 3D printing has developed rapidly. Metal 3D printing facilitates the manufacture of complex geometrical and multifunctional parts and continues to meet the clinical needs of enhanced osteointegration.

### 4.2. Bioceramic Materials

Bioceramics are divided into biodegradable ceramics and nonbiodegradable ceramics. The former includes hydroxyapatite (HA) and calcium phosphate (CaPs), and the latter includes zirconia and alumina [55]. 3D printing porous ceramics is conducive to meeting patients' needs for lightweight and multifunctional materials. HA is similar to the inorganic components of bones and teeth. It has good biocompatibility, bone conduction, osteoinduction, and degradability. It is often used for bone defect repair and is the most widely used artificial bone substitute material. The PGA scaffold containing HAp nanoparticles was prepared by 3D printing technology, and the bone regeneration rate was 47% 8 weeks after surgery. Compared with the existing scaffolds, the porous PGA/HAp scaffold was implanted in the rabbit skull defect model, and the key defect area was covered by bone tissue, which indicates that the scaffold can promote the bone regeneration process [56]. Due to the nondegradability, good wear resistance, and low thermal conductivity of zirconia through 3D printing, it is widely used in ceramic abutments, implants, and crowns, as well as the femoral head part of hip prostheses in THA [57]. High-purity alumina has biological inertness and a high surface finish and is often used in the parts of pacemaker pumps. Combining 3D printing technology with liquid precursor infiltration, through controlling the number of infiltrations and sintering temperature, aluminum toughened zirconia (ATZ) can be obtained, with good hardness and fracture toughness [58]. This is conducive to the manufacture of complex ultrafine shapes and has potential application value in the field of orthopedics.

### 4.3. Polymer Materials

Polymer materials are divided into natural and synthetic materials. Natural materials mainly include chitosan, polylactic acid (PLA), collagen, and hyaluronic acid. Synthetic materials mainly include polycaprolactone (PCL), hydrogel, and polyether ether ketone (PEEK). Chitosan has good biodegradability, biocompatibility, and anti-inflammatory activities and is widely used in wound healing, bone and cartilage tissue engineering, and drug delivery [59, 60]. Studies have shown that chitosan can promote the secretion of proinflammatory cytokines, has an inhibitory effect on Escherichia coli and Staphylococcus aureus, and exhibits antibacterial activity [61, 62]. Therefore, we can use chitosan to modify the scaffold to prevent inflammatory effects caused by the implant. However, the mechanical properties of chitosan scaffolds are poor and easy to deform. It is necessary to explore methods of compounding with other materials to manufacture scaffolds that meet clinical needs. Research by Fairag et al. showed that polylactic acid (PLA) promotes the proliferation and differentiation of osteoblasts and promotes the production of the bone-like matrix. PLA scaffolds are used as bone substitutes to repair bone defects. Due to its biodegradability and biocompatibility, PLA is widely used in surgical sutures, orthopedic screws, and fixation materials [63]. The main factor limiting the application of PLA is its low affinity with cells, so it is necessary to find suitable modified materials to enhance its hydrophilicity.

PEEK has an elastic modulus close to that of human bone and is an ideal orthopedic implant material. PEEK is used in intervertebral fusion cages and skull repair [64]. However, the lack of cell adhesion recognition sites on the surface of PCL and PEEK leads to cell loss during bone regeneration. Explore different materials to manufacture tissue engineering scaffolds to meet the requirements of implantation in the human body. Recent studies have shown that a functionally graded scaffold (PCL-*β*-TCP FGS) made of polycaprolactone and *β*-tricalcium phosphate can significantly increase bone ingrowth in the bone tunnel, providing a new idea for the early treatment of femoral head necrosis [65]. Kruse et al. [66] processed the PEEK scaffolds by plasma immersion ion implantation (PIII), which provided high-density free radicals on the surface, improved hydrophilicity, and achieved strong adhesion of the mineralized layer. The PIII method is used to improve the surface biological activity of the PEEK scaffold and improve the bone binding ability of the scaffold. The high water content and mechanical characteristics of hydrogels are similar to those of the extracellular matrix, which are used to simulate the in vivo environment and communicate information between cells. The interaction of hydrogel adhesion forces is a major challenge. It has been proved that the introduction of cellulose nanofibers and nanocrystals into hydrogels can simulate the natural characteristics of the human body's weight-bearing and electroactive tissues [67].

### 4.4. Composite Materials

**Figure 5:**
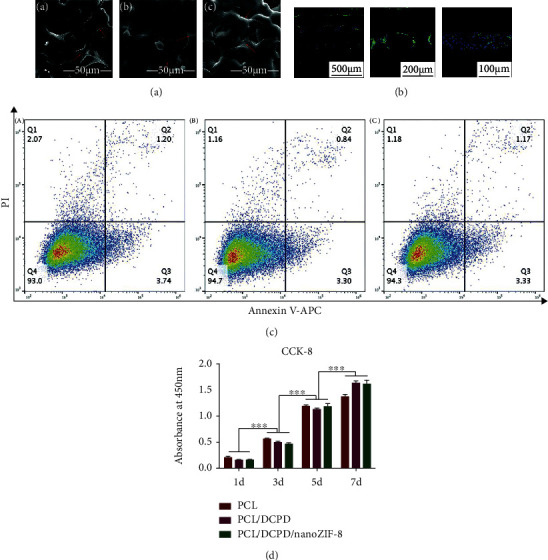
Biocompatibility of the 3D printed scaffolds seeded with BMSCs. (a) Morphology of the cells seeded on the surface of the scaffolds observed by SEM: (A) PCL, (B) PCL/DCPD, and (C) PCL/DCPD/nanoZIF-8. Arrows point to the BMSCs adhering to the surface of scaffolds. (b) Representative images of cell spatial distribution on PCL/DCPD/nanoZIF-8 scaffolds using LSCM. (c) Cell apoptosis rate examined by Annexin V assay: (A) PCL, (B) PCL/DCPD, and (C) PCL/DCPD/nanoZIF-8. (d) Cell viability measured by CCK-8 assays on days 1, 3, 5, and 7. Significant differences were marked among a series of points for each group, respectively. Data were shown as mean ± SD, ^∗∗∗^*p* < 0.001 (*n* = 4) [70]. Used with permission from the Royal Society of Chemistry.

In clinical application, the above materials have different advantages and disadvantages. Therefore, different types of materials need to be integrated to meet the needs of implants. Composite materials are made of two or more different types of materials. The combination of ceramics and metal materials, as well as ceramics and polymer materials, has become a new breakthrough in the application of 3D printing materials. Fernández-Cervantes et al. constructed a polylactic acid/sodium alginate/hydroxyapatite composite scaffold. The density and microporosity of this scaffold material are close to natural bone tissue, and the compressive strength value is greater than the maximum range of trabecular bone. It is suitable as a bone scaffold in tissue engineering [68]. Zhang et al. modified the *β*-tricalcium phosphate scaffold with silver nanoparticles and graphene oxide. Through the antibacterial test of gram-negative bacilli, it was found that the scaffold had good antibacterial activity; by increasing the alkaline phosphatase activity and bone-related gene expression in rabbits, it was found that this scaffold could promote osteogenic differentiation [69]. This is of great significance for clinical research to reconstruct bone defects while preventing infection. The PCL/DCPD/nanoZIF-8 porous composite scaffold constructed by Zhong et al. using extrusion 3D printing technology has good bionic structure and mechanical properties. In vivo and in vitro studies have shown that the scaffold can control the release of calcium and zinc ions and promote the differentiation of bone marrow mesenchymal stem cells into osteoblasts [70]. As shown in Figure 5, nanoZIF-8 as a metal-organic framework incorporated into a 3D printed complex scaffold has good biocompatibility, increases the proliferation activity of bone marrow mesenchymal stem cells, promotes cell adhesion and proliferation, and promotes new growth in the body, which is of great significance for the treatment of bone defects.

### 4.5. Performance Requirements of 3D Printing Materials

In the 3D printing process, printing materials should be selected and designed according to actual application requirements and material characteristics. 3D printing needs to consider the material's biocompatibility, biodegradability, printability, and mechanical stability. Printability requires accurate control of material processing performance on time and space scales during the printing process to ensure product structure and dimensional accuracy [55]. Different printing technologies have different requirements for printability. For example, inkjet printing requires materials to have fast cross-linking characteristics, which facilitates the layered molding of complex 3D structures to form a stable structure [55]. The printing material should have a certain degree of ability to withstand external forces so as to maintain the function of the printed product. However, natural polymer materials have poor biomechanical properties, so other materials must be selected as support materials when used in 3D printing [71]. Tissue engineering scaffolds need to consider the biodegradability of materials. The rate of material degradation matches the rate of cell growth and proliferation. The degradation products are nontoxic to the body and are rapidly excreted through metabolism.

The existing 3D printing technology cannot meet all clinical needs, so it is necessary to combine other materials and methods to improve the performance of the materials and meet the clinical needs. 3D printed products have poor surface roughness, curvature, and other surface properties, which do not meet the requirements of orthopedic implants. Therefore, it is necessary to use surface coating modification and other further processing before implantation to obtain a good surface finish. Electrochemical polishing (EP) posttreatment has great potential in improving the surface performance of the stent. Habibzadeh et al. treated 316L stainless steel with EP, which increased its biocompatibility and improved the surface properties of implant materials [72, 73].

A table (Table 2) is presented to summarize the advantages and disadvantages of additive manufacturing materials.

## 5. Application of 3D Printing in Orthopedic Smart Implants

As implantable medical devices, orthopedic smart implants play an important role in the diagnosis and treatment of diseases. Smart implants can be used to continuously monitor changes in the internal environment of the patient's body to achieve “early detection, early diagnosis, and early treatment,” minimize disease complications, and save social medical and health resources. The development of medical telemetry technology and wireless sensors provides a new opportunity for the implantation of tiny sensors and orthopedic devices in the body [86]. At present, there have been reports on the application of intelligent orthopedic implants in bone healing assessment, knee joint force analysis, spinal fusion monitoring, hip prosthesis loosening monitoring, and so on [87]. Patients with advanced avascular necrosis of the femoral head (ANFH) and femoral neck fracture usually require total hip arthroplasty (THA), which involves resection of the femoral head and proximal femur and acetabular surface replacement. Prosthesis loosening is one of the common complications of THA. Mechanical magnetic sensors (also called oscillators) can early identify the osseointegration of the implant for the early diagnosis of prosthesis loosening [88]. The oscillator is placed in the femoral shaft, the coil outside the human body is set to activate the internal oscillator, the speed of the vibrator hitting the film is detected by another coil outside the body, and the postprocessing determines whether the prosthesis is loose. The main challenges facing total knee arthroplasty include the optimal pressure for the placement of the prosthesis and how to balance the pressure of the surrounding soft tissues and collateral ligaments. At present, this all depends on the surgeon's clinical experience. The use of wireless piezoresistive stress sensors to measure the pressure of knee implants in real time [89] helps to solve this problem.

With the advancement of 3D printing technology and materials, the manufacture of multilayer and multimaterial (MLMM) electronic devices can be realized. The hybrid printing method is used to combine different printing technologies to realize the preparation of complex structure MLMM. Hoerber et al. [90] combined aerosol jet technology and powder bed fusion to provide an opportunity for the functionalization of 3D printed MLMM electronic devices, such as installation and insertion of electronic components. Combining different 3D printing technologies, through the embedding of electronic components, the connection of circuits between layers, and the alternating printing of insulating and conductive materials, the 3D printing of multilayer circuits is realized [91].

## 6. Challenges and Future Perspectives

### 6.1. Poor Bionic Effect

At this stage, 3D printing can construct single-function tissues, but it is still very difficult to construct organs with complex structures and satisfying physiological functions. Information transmission between cells usually relies on the nerve and blood vessel network. However, the neurovascular network of the printed organs in the in vitro culture phase cannot grow in a short period of time, resulting in a lack of oxygen, nutrients, bioactive factors, and waste accumulation, resulting in necrosis of the implanted tissue [55]. Therefore, we need to develop ideal printing technology to solve the problem of bionic structure. First of all, it is necessary to continuously improve printing speed and ink compatibility to achieve high-precision printing. For example, combining traditional electrospinning with 3D printing technology has improved the resolution of the printing platform [25]. Second, in order to realize the complex structure and function of printed organs, a variety of different 3D printing technologies can be combined in the same device so that different materials and technologies are combined. New technologies continue to be combined with 3D printing technology. For example, acoustophoretic drop-on-demand patterning printing expands the viscosity by four orders of magnitude while ejecting microliter- or nanoliter-volume droplets [92]. Li et al. used heat-responsive hydrogels to construct a tiny vascular system. The results showed that the number of blood vessels around the stent increased and the host's blood vessels grew in, which indicated a new direction for the bionic strategy.

### 6.2. Safety and Effective Supervision of 3D Printing

As 3D printing is increasingly used in the medical field, the quality assurance of implants is more important. Photopolymerizable resins and photoinitiators trigger the polymerization reaction by converting them into active free radicals. Uncured resins may contain high concentrations of free radicals, which may cause cancer [6]. The lattice structure provides a suitable surface for microbial adhesion, so implant-related infection is another issue to be considered. The toxicity associated with degradation is another challenge that must be considered. Excess metal ions have cytotoxicity and genotoxicity. For example, Al and V in the Ti6Al4V porous scaffold are cytotoxic. The powder used in 3D printing makes it easy to absorb moisture, oxidize, and even pollute. How to maintain the purity of the powder in the additive manufacturing process is very important [93]. Constantly explore new materials and material modification methods to increase biocompatibility. Relevant departments should build a closed-loop quality control system that combines planning, monitoring, and feedback control and continuously improve laws and regulations related to AM industry quality assurance standards.

### 6.3. Lack of Ideal Bioinks

The selection of bioink materials mainly considers the biocompatibility, mechanical properties, and degradation time of the materials. The current bioink is mainly composed of living cells and bioactive polymers. The patient's autologous cells are very limited, and allogeneic cells are prone to the risk of immune rejection and pathogen infection. Traditional hydrogels are randomly cross-linked single-network hydrogels, and increasing the cross-linking density and polymer content to enhance the hydrogel network will inevitably reduce the permeability and porosity required for cell growth, thus affecting the cultivation of the cell [94]. With the further exploration of materials and preparation methods, it is the future development trend of hydrogels to respond to stimuli such as external temperature and pressure. In recent years, stem cells have become a research hotspot due to their ability to induce differentiation into cells with different functions. Nakamura et al. used neural crest cells derived from human induced pluripotent stem cells (iPSC) to produce cartilage through mesenchymal stem cells that have the potential to differentiate into cartilage. After 3 weeks, they found changes in the expression of collagen II. This research provides a solution for transplant immune rejection [95]. In addition, with the breakthrough of biomaterials and nanotechnology, bioinks that meet bioactivity and large-scale cultivation will continue to be explored.

## 7. Conclusions and Prospects

As 3D printing technology begins to be applied to the medical field, it is important to understand the characteristics of 3D printing materials. In orthopedics, 3D printing materials can be made into implants, prostheses and creation of life-size anatomical models. With the further improvement of 3D printing technology and the reduction of printing cost and image postprocessing time, 3D printing will be included in routine clinical diagnosis in the near future. Among the various types of 3D printing systems used in biomedicine, most previous studies have focused on FDM printing systems, and polymer filaments are the main raw material for bone preparation. This method has many advantages in three-dimensional bioprinting but is limited by its principle and can only print metal and plastic nonbioactive materials. One of the important goals of 3D printing research in the field of tissue engineering is to develop ideal 3D printing materials that have both good biocompatibility and high printing performance. Biocompatible materials are also used to enhance and promote bone regeneration in orthopedics. In short, 3D printing is a groundbreaking technology and has matured.

In recent years, 3D printing technology, as an emerging interdisciplinary subject, is closely integrated with materials science, clinical medicine, and biology, providing new treatment strategies for precision medicine. 3D printing is widely used in orthopedics: 3D printing models are used for preoperative education, printing osteotomy guides, making personalized rehabilitation braces, and treating bone defects. With the advancement of materials, 3D printing systems are developing in the direction of scalable nanomanufacturing. However, due to the inherent competition between printing speed and printing resolution, macroscopic polymer objects with nanoporosity are currently impossible to directly print in three dimensions. New 3D printing materials may achieve this change in the future. An orthopedic smart implant is an implantable medical device, but its clinical application is restricted due to barriers to entry. With the continuous progress of 3D printing MLMM electronic devices, people are rethinking the manufacturing methods of electronic components and equipment. Before smart implants are widely used in clinical practice, there are still many difficulties that need to be resolved. Continuously modify implants to adapt to electronic devices and sensors while miniaturizing and intelligentizing wireless sensors, thereby improving the performance of orthopedic implants. In addition, 3D printing still has some problems. 3D printing only considers the initial state of the printed object, ignoring the dynamics of creatures. Implantation in the body cannot adapt to changes in the internal environment and cannot achieve the expected results. With the continuous in-depth research on 3D printing materials, responsive materials that can respond to stimuli such as temperature and pressure are combined with 3D printing technology so as to realize that the structure and function of 3D printing objects change with changes in the internal environment. This article reviews the main technologies and materials of 3D printing in the field of orthopedics, discusses a series of challenges currently faced by 3D printing, and looks forward to the development of 3D printing. In summary, based on our findings, 3D printing technology has the potential to become a powerful tool for personalized and effective treatment in orthopedics.

## Figures and Tables

**Table 1:** Summary of 3D printing techniques.

3DP techniques	Materials	Advantages	Disadvantages	References
Vat photopolymerization	Photopolymers, ceramics, composites	(i) High building accuracy (ii) Fast building rate (iii) Excellent part quality	(i) High shrinkage (ii) Mechanical failure including curling and warping	[34–36]
Directed energy deposition	Metals, ceramics, polymeric, composites	(i) Supporting a wide range of materials (ii) Fabrication of structurally graded materials with differing porosities (iii) Allow the manufacture of tailored structures in the material	(i) Residual stress (ii) The surface roughness of the fabricated part (iii) Postprocessing is often tedious and increases the lead time and the cost	[7, 37, 38]
Binder jetting	Ceramics, metals	(i) Low cost (ii) Suitable mechanical performance (iii) Safe material	(i) Low resolution output (ii) Not easy to operate (iii) Cannot apply heat sterilization (iv) Excess powder will contaminate the part	[2, 39]
Fused deposition modeling	Thermoplastic polymers, composites, low-melting temperature metal alloys	(i) Simplicity (ii) Cheap cost and easy (iii) Accessibility (iv) Filaments are cheap and arrive in various colors (v) Fundamental for thinner layers up to 0.1 mm thick	(i) Thermal degradation of ingredients (ii) Discontinuous extrusion results in the formation of defects (iii) Slow drug dissolution speed (iv) The seam between layers is visible (v) Poor adhesion results in delamination, causing pores and defects and reducing mechanical properties (vi) FDM requires thermoplastic polymers, and most pharmaceutical grade polymers are not thermoplastics	[40–42]
Powder bed fusion	Polymers, ceramics, metals	(i) Components exhibit excellent mechanical properties (ii) Support structures are not required in the SLS process (iii) Produce high-quality metal components that are free of internal stresses using a wide range of metal powders	(i) An expensive process (ii) EBM requires vacuum operation (iii) The SLS process can use only plastic powders as raw materials (iv) Material waste is relatively high, and harmful gases are released during fabrication in the SLS process	[5, 43, 44]

**Table 2:** Summary of additive manufacturing materials: advantages and disadvantages.

Material types	Composition	Advantages	Disadvantages	Ref.
Metal	Titanium alloy, copper alloy, Ti6Al4V alloys, CoCrMo alloys	(i) Good toughness (ii) Antibending fatigue performance (iii) Biocompatibility (iv) Excellent processing performance (v) Good corrosion resistance (vi) Easy to manufacture expensive materials with complex geometries or materials that are difficult to process	(i) Pure titanium easily leads to a stress barrier (ii) Potential biological safety; excessive metal ions such as Zn^2+^ show cytotoxicity (iii) The use of metals may lead to some rare consequences, such as allergies or genotoxicity (iv) Poor adhesion on the surface of inert implants such as titanium alloys (v) Metal stent printing needs to be performed under high-temperature conditions, and the stent printing cannot be synchronized with the coating of biologically active molecules or mixed printing of cells	[7, 55, 74–76]
Bioceramics	Hydroxyapatite (HA), calcium phosphate (CaPs), tricalcium phosphate, MgP, alumina, zirconia	(i) Good biocompatibility (ii) Biodegradability (iii) Strong resistance to pressure (iv) Achieve very high structural resolution (v) Strong osteoinductive ability (vi) Alumina and zirconia have good toughness and wear resistance and high mechanical strength (vii) Alumina and zirconia's low thermal conductivity and nondegradability	(i) Ceramic stents need to be printed at high temperatures, and stents cannot be simultaneously coated with bioactive molecules that promote bone formation or anti-infective drugs (ii) High brittleness (iii) Poor toughness (iv) Weak shear stress	[77–79]
Polymer materials	Polycaprolactone, polylactic acid-glycolic acid, polyglycolic acid, collagen, alginate, silk fibroin, chitosan	(i) Biocompatibility (ii) Biodegradability (iii) Good thermoplasticity (iv) Natural polymer material with natural porous structure and good hydrophilicity (v) Increasing the roughness of the scaffold can improve the adhesion ability of cells	(i) Natural polymer materials are difficult to obtain in large quantities, degrade quickly, and have insufficient biomechanical strength (ii) Viscosity-dependent fluidity of polymers (iii) Biodegradation will produce lactic acid and carbon dioxide, and the local pH will decrease, thereby creating an acidic environment that is conducive to inflammation rather than healing (iv) The lowering of the pH value of the local environment can accelerate the hydrolysis of the ester bond of the polymer material, promote the degradation of the polymer material, and affect the biomechanical effect of the stent (v) The polymer scaffold printed with cells has weak resistance to compression and cannot meet the compression requirements of human bones	[80–83]
Composite materials	Polylactic acid-glycolic acid; copolymer/tertiary calcium phosphate; PCL/DCPD/nanoZIF-8; polycaprolactone mixed with *β*-tricalcium phosphate	Combined with the advantages of the above materials		[55, 70, 84, 85]
